# Angle-torque relationship of the subtalar pronators and supinators in younger and elderly males and females

**DOI:** 10.1186/s13047-015-0125-2

**Published:** 2015-11-24

**Authors:** Marco Hagen, Daniel Sanchez-Bergmann, Sebastian Seidel, Matthias Lahner

**Affiliations:** Biomechanics Laboratory, Department of Sport and Movement Sciences, University of Duisburg-Essen, Gladbecker Str. 182, 45141 Essen, Germany; Department of Orthopaedic Surgery, Ruhr-University Bochum, St. Josef-Hospital, Gudrunstr. 56, 44791 Bochum, Germany

**Keywords:** Subtalar joint angle, Muscle strength, Pronators, Supinators, Age, Sex, Range of motion

## Abstract

**Background:**

The angle-dependent torque capacity of the subtalar pronators and supinators is important to maintain dynamic ankle stabilisation. Based on the peak torques during maximum voluntary isometric pronation and supination across the subtalar range of motion, the strength curves of younger and elderly males and females were investigated.

**Methods:**

Maximum voluntary isometric subtalar pronator and supinator strength tests were administered to 30 younger and 30 elderly volunteers (each 15 male and 15 female subjects). Total active subtalar range of motion and peak pronator and supinator torques were measured in five anatomical subtalar joint angles using a custom-built apparatus with two force transducers. Furthermore, relative torques (normalised to the individual peak torque) and pronator-to-supinator strength-ratios were also calculated.

**Results:**

Pronator-to-supinator strength ratio, and peak pronator and supinator torques are affected by age and by joint angle x age interactions. All supinator strength curves show a steadily descending characteristic from the pronated to the supinated positions. The pronator strength curve had an inverted U-shaped characteristic, except for younger women of whom 47 % exert highest peak values in the end-range pronation angle. Both relative pronator and supinator strength are dependent on sex (*P* < 0.05). Relative pronator strength is also affected by joint angle x sex (*P* < 0.0001) and joint angle x sex x age (*P* < 0.05) interactions. Beside age effects on all range of motion parameters, pronation range of motion is influenced by a sex x age interaction (*P* < 0.05).

**Conclusions:**

Age- and sex-related differences in both subtalar strength profile and range of motion have to be considered when testing strength across subtalar range of motion. Younger females have higher pronator strength capacity in the most pronated joint angle, which may be due in part to their greater subtalar joint range of motion compared to the other groups. In the most supinated position both pronator and supinator strength capacity is reduced in younger females compared to younger males.

**Electronic supplementary material:**

The online version of this article (doi:10.1186/s13047-015-0125-2) contains supplementary material, which is available to authorized users.

## Background

The muscular capacity of the subtalar pronators and supinators of the foot plays a key role in the medio-lateral stability of the ankle joint complex [1–3]. In the prevention of recurrent ankle sprains, subtalar joint-specific pronator strength training is recommended to counteract peroneal muscle weakness [4, 5], to enhance pronator-to-supinator strength-ratio [6, 7], and to regulate inappropriate foot positioning before ground contact [8]. Strengthening the supinators increases the antipronator capacity of the deep plantarflexors (tibialis posterior, flexor hallucis longus and flexor digitorum longus) [2] which is potentially beneficial in the prevention of running-related overuse injuries [9, 10].

It is well known that the human capability to exert torque around a given joint varies throughout the range of motion. This so-called ‘strength curve’ is explained, firstly, by changes in the length of each muscle surrounding the joint, the length-tension relationship [11]. Secondly, the length of the lever arms varies when the joint position is changed. Due to anatomical differences, the strength curve of each joint has its individual shape [12]. Although isokinetic testing is often applied in strength diagnostics, Hay [11] recommends repeated maximum voluntary isometric contractions (MVIC) in different joint angles for determining a strength curve. Isokinetic measurements show remarkable variability at the extreme ranges of the movement, because isokinetic dynamometers do not provide a constant angular velocity across the full range of motion [13].

So far, there has been little research into the angle-dependent strength capacity of the pronators and supinators. As pronator and supinator muscle strength is important to counteract inversion and eversion moments [14], the isometric strength capacity should be assessed across a wider range of subtalar motion. Due to the complex geometry of the subtalar joint with an oblique resultant movement axis [15], the strength curves of the pronators and supinators of the foot are poorly understood. Although research has focused the peak torques during isokinetic strength measurements, our recent study is the only one which investigated maximum voluntary isometric pronator and supinator strength across the active range of subtalar joint motion. In a sample of healthy young males, the pronator strength curve showed an inverted U-shaped characteristic, whereas the supinator curve descends from pronated to supinated position [16]. Apart from the peak torques, the pronator-to-supinator strength ratio (PSR) is clinically relevant because co-contraction of opposing muscles across a joint is important to maintain dynamic joint stability [17]. It is well known that both active and passive properties of skeletal muscle are influenced by age [18, 19] and sex [20–23]. It is hypothesized that sex- and age-related differences in muscle strength influence the pronator and supinator strength curve characteristics and the PSR. Hence, the purpose of the present study was to investigate the isometric angle-dependent pronator and supinator strength capacity in younger and older males and females.

## Methods

### Participants

Maximum isometric strength tests of the pronators and supinators of the dominant foot were administered to 30 younger and 30 elderly volunteers (each 15 male and 15 female) (Table 1). The younger participants were sports students of the local university, and the elderly were recruited by word-of-mouth recommendation and by postings at the local university. All volunteers completed a screening health questionnaire before participation. All participants reported no contraindications to resistive exercise, no major neuromusculoskeletal dysfunction of the lower extremities, and no orthopaedic, cardiac or visual problems in the past two years. No medication was being taken by the subjects that would have been expected to affect physical performance. All elderly people lived self-determined without extra-care. The navicular drop test was performed by an experienced clinician (DSB) to determine the characteristics of the medial longitudinal foot arch. As increasing midfoot mobility might have confounded the outcome measures, participants having greater than 10 mm of navicular drop [24] were excluded from the study. This criterion has previously been used to classify participants as having excessive pronation [25, 26]. Background information and informed written consent was collected prior the first test session. The study was approved by the ethics committee of the local university hospital in accordance with the Helsinki Declaration.

**Table 1:** Anthropometric data (mean ± SD)

Group (n)	Age (years)	Height (m)	Body mass (kg)	Body mass index (kg/m^2^)	Foot length (mm)	Navicular drop (mm)
Young women (15)	23.9 ± 2.1	1.71 ± 0.07	62.9 ± 7.9	21.4 ± 2.2	24.6 ± 0.9	49 ± 12
Young men (15)	26.3 ± 2.7	1.85 ± 0.07	84.2 ± 10.8	24.7 ± 2.7	27.7 ± 1.0	44 ± 12
Elderly women (15)	66.7 ± 6.1	1.63 ± 4.7	68.6 ± 12.4	27.0 ± 4.5	24.6 ± 1.1	40 ± 18
Elderly men (15)	61.5 ± 5.4	1.78 ± 8.0	85.3 ± 13.0	25.7 ± 4.9	26.8 ± 1.2	48 ± 16

### Instrumentation

**Fig. 1:**
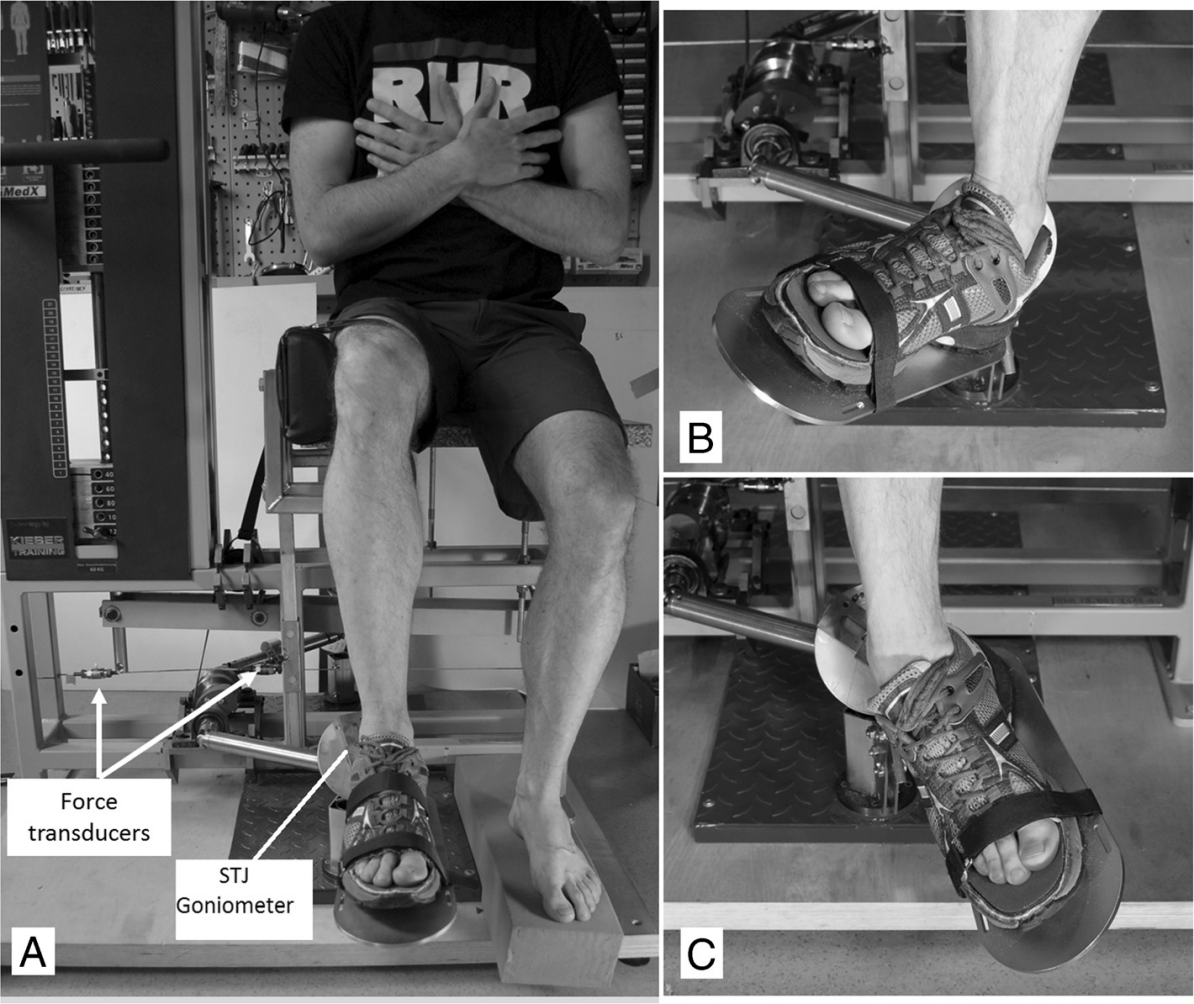
Biomechanical setup for determination of the isometric angle-torque relationship of the subtalar pronators and supinators. **a** Neutral position; **b** -24° pronated position; **c** 40° supinated position

Subtalar strength testing was performed using a specific foot apparatus mounted on a wooden base plate [16] (Fig. 1). The movement axis was orientated corresponding to the subtalar joint axis deviating about 23° to medial and about 41° to dorsal from the longitudinal foot axis [15]. A sport shoe (males: size US 10; females: size US 7) was mounted onto the foot plate. The toe box of the shoe’s upper was removed so that strength testing was possible for subjects with variable foot lengths between 24.5 cm to 25.7 cm (i.e. shoe sizes US 6 to 8) for females and between 26.5 cm to 29.5 cm (i.e. shoe sizes US 8.5 to 11.5) for males. In neutral position (shank perpendicular to foot sole), the foot plate of the apparatus was aligned in parallel to the floor and to the longitudinal axis of the foot. During testing, the forefoot was additionally fixed with a belt. To eliminate the mechanical influence of gastrocnemius muscle, which would have influenced the range of ankle and subtalar joint motion as well as the resulting pronator and supinator capacity, strength testing was performed in a seated position so that hip and knee joint were each positioned at approximately 90°. Associated motions of hip and knee were prevented by straps which were placed around the thigh (Fig. 1).

Two force transducers (Kistler 9321A, Winterthur, Switzerland) were used to record the maximum resulting voluntary isometric pronator and supinator torques. According to the assumption that the axis of the foot apparatus corresponds to the subtalar joint axis [15], strength testing was administered in random order in five anatomical positions within the subtalar movement plane: 24° and 8° pronated position, 8°, 24° and 40° supinated position (Fig. 1). The angles are related to neutral position with the shank perpendicular to horizontal and the foot (2^nd^ ray) oriented in parallel to the thigh (Fig. 1a) so that the frontal plane angle between the calcaneus and the tibia was 0°. Degrees of pronation were negative, and degrees of supination were positive. The correct placement of the anatomical angle was controlled by using an electrogoniometer (Megatron MP 10, Germany) which was aligned with the machine axis.

### Strength testing

As isolated subtalar pronation and supination are quite unfamiliar movements, the participants were instructed in the setup approximately one week before the experiment started. After verbal explanation and a practical demonstration, the participants performed a number of practice strength tests with their dominant foot. Leg dominance was determined as the leg which is preferred for kicking a ball. On the experimental day subjects underwent a 10-min warm up on a bicycle ergometer and a number of submaximal repetitions for familiarisation. As described previously [27], all subjects performed three valid maximum voluntary isometric pronation and supination contractions in each subtalar joint angle. In each trial the participants were instructed to perform a ramp contraction and to hold MVIC for a minimum of two seconds. Rapid contractions showing an initial spike were excluded from the analysis and repeated. Strength testing was supplemented by biofeedback procedure by displaying the real time signal of the force sensor on a monitor. All experimental applications were conducted by the same testers (DSB, SS).

The maximum of each torque-time-curve was registered as peak pronator torque (PPT) and peak supinator torque (PST), respectively. Before each MVIC trial, the force sensor signal was reset to zero. Thereby, the effect of gravity on the combined weight of the leg and the movement arm of the foot apparatus as well as the pretension of the passive structures were eliminated. Thus, it can be assumed that only the active torque-length-relationship of the pronators and supinators of the subtalar joint was determined. A two-minute rest was provided between the trials to prevent fatigue [28]. Apart from the PPT and PST normalized to body mass, the relative strength curves were analysed. For this purpose the PPT and PST data were normalized by setting a value of 100 to the peak value of each subject’s torque-length-relationship and expressing the values obtained at the other joint angles as a percentage of the peak value. Furthermore, the PSR was calculated by dividing the PPT to the PST for each joint angle.

### Range of subtalar motion

Before strength testing, the active range of subtalar motion (ROM) was quantified using the procedure by Hagen et al. [29]. The subjects performed three repeated pronations and supinations to maximum end-range of active motion. Maximum pronation and supination ROM and the overall ROM (sum of pronation and supination ROM) were determined.

### Statistical analysis

The measurement parameters were averaged for each subject before further statistical treatment. Unless otherwise stated, an alpha level of 0.05 was set for significance for all statistical analyses. A three-way ANOVA with repeated measures comprising ‘joint angle’ and the independent factors ‘age’ and ‘sex’ was applied to identify differences in angle-torque relationship and the relative strength curves. According to the relative pronator and supinator strength curves, post-hoc tests were conducted with subsequent Bonferroni corrected significance to 0.0083. A chi-square test was used to determine if there were significant differences among groups in the joint angle at which the peak of the strength curve occurred. A two-way ANOVA was performed to identify ROM differences between factors ‘age’ and ‘sex’.

## Results

In Table 2 the differences in subtalar ROM are presented. The ANOVA reveals a significant age x sex interaction (*P* < 0.05; F_(1,56)_ = 4.9; η^2^_p_ = 0.08), indicating that younger females have greater pronation ROM compared to the other three groups. Significant main effects of age on supination (*P* < 0.05; F_(1,56)_ = 5.8; η^2^_p_ = 0.09), pronation (*P* < 0.01; F_(1,56)_ = 8.1; η^2^_p_ = 0.13) and overall ROM (*P* < 0.01; F_(1,56)_ = 10.5; η^2^_p_ = 0.16) reveal reduced ROM in the elderly groups.

**Table 2:** Range of motion (mean ± SD)

Group (n)	Pronation ROM (°)	Supination ROM (°)	Overall ROM (°)
Young women (15)	47.5 ± 6.5	53.9 ± 6.6	101.4 ± 7.9
Young men (15)	41.9 ± 4.1	52.3 ± 8.2	94.2 ± 10.8
Elderly women (15)	40.3 ± 6.3	48.7 ± 5.8	89.0 ± 10.4
Elderly men (15)	41.0 ± 4.8	49.0 ± 6.8	90.0 ± 10.4
P-Values (2-way-ANOVA)	<0.01^a^	<0.05^a^	<0.01^a^
	<0.05^b^		

^a^significant main effect: age

^b^significant interaction: age x sex

*ROM* range of motion

**Fig. 2:**
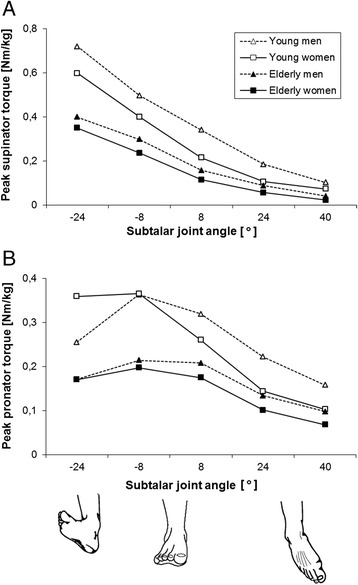
Peak supinator (**a**) and pronator torque (**b**) normalised to body mass. Mean values are shown without error bars for clarity

In Fig. 2, the angle-dependent PSTs and PPTs (normalized to body mass) are illustrated. Across all groups the PST curves show a steadily descending shape from the pronated to the supinated joint angles (Fig. 2a). In contrast, the PPT curves have an ascending-descending characteristic except for the younger females who do not show increasing torque between −24° and −8° (Fig. 2b). Both PPT and PST are affected by significant joint angle x age interactions (PPT: *P* < 0.001; F_(4,56)_ = 8.29; η^2^_p_ = 0.13; PST: *P* < 0.0001; F_(4,56)_ = 22.4; η^2^_p_ = 0.29). Apart from significant main effects of joint angle for PPT (*P* < 0.0001; F_(4,56)_ = 75.55; η^2^_p_ = 0.574) and PST (*P* < 0.0001; F_(4,56)_ = 371.8; η^2^_p_ = 0.87), both PPT (*P* < 0.0001; F_(1,56)_ = 38.55; η^2^_p_ = 0.41) and PST (*P* < 0.0001; F_(1,56)_ = 63.9; η^2^_p_ = 0.53) are higher in younger compared to elderly people. As indicated by the joint angle x age interactions, these age-related differences in PST and PPT depend on the subtalar joint angle. The ANOVA also reveals a significant main effect of sex (*P* < 0.01; F_(1,56)_ = 12.5; η^2^_p_ = 0.183) on PST.

**Fig. 3:**
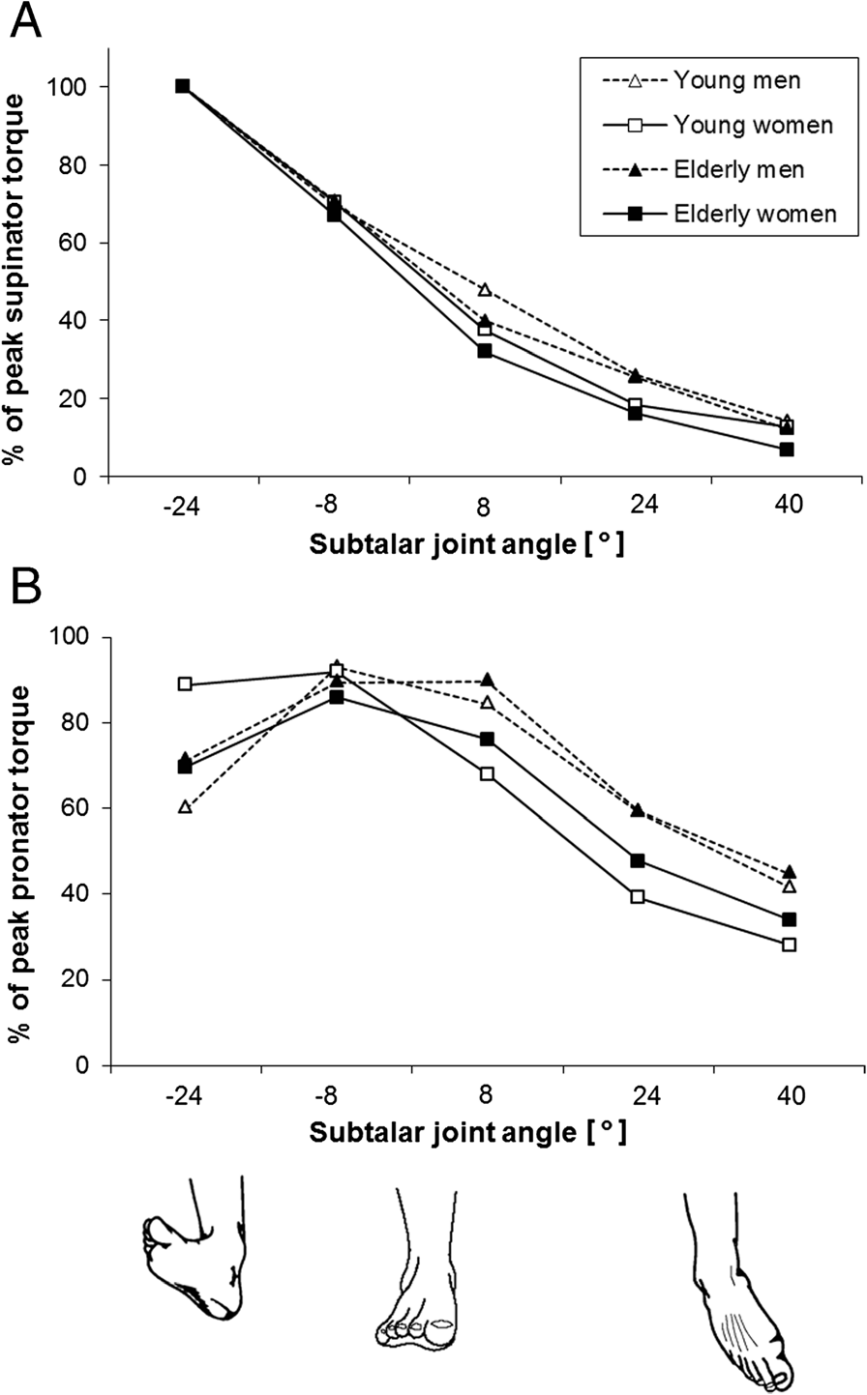
Relative supinator (**a**) and pronator torque (**b**) as a percentage of peak torque throughout the range of subtalar motion. Mean values are shown without error bars for clarity

The relative supinator strength curves (Fig. 3a) show a nearly congruent characteristic between −24° and −8°. There is a trend to a joint angle x sex interaction (P = 0.05; F_(4,56)_ = 2.4; η^2^_p_ = 0.04) indicating a higher angle-dependent supinator strength capacity for males which becomes obvious in joint angles 8° (+26 %) and 24° (+50 %). Apart from a significant main effect of joint angle (*P* < 0.0001; F_(4,56)_ = 742.5; η^2^_p_ = 0.93), the ANOVA reveals a significant main effect of sex (*P* < 0.01; F_(1,56)_ = 7.5; η^2^_p_ = 0.12) on relative supinator strength. In joint angle 8°, the post-hoc analyses reveal significant differences (*P* < 0.01) between the younger males and the other three groups.

The pronator strength curve of the younger females differs from the other groups (Fig. 3b). Both male groups and the elderly females display an inverted U-shaped pronator strength curve with increasing strength from −24° to −8°. In the younger males and both female groups the curve descends from −8° to 40°, while in elderly males a plateau of peak pronator strength is present around the neutral subtalar joint position between −8° and 8°. In contrast, relative peak pronator torque of young females is nearly equal in −24° and −8° with 89 and 92 %, respectively. Interestingly, in joint angle −24° relative pronator strength is 58 % higher in younger females compared to younger males (*P* < 0.01). According to the differences in the relative pronator strength, the ANOVA reveal significant joint angle x sex x age (*p* < 0.05; F_(4,56)_ = 2.9; η^2^_p_ = 0.05) and joint angle x sex (*P* < 0.0001; F_(4,56)_ = 6.4; η^2^_p_ = 0.10) interactions. Significant main effects of joint angle (*P* < 0.0001; F(4,56) = 79.9; η^2^_p_ = 0.59) and sex (*P* < 0.01; F(1,56) = 10.6; η^2^_p_ = 0.16) on relative pronator strength were found. In joint angle 40°, significant differences become obvious between younger females and both younger (*P* < 0.01) and elderly males (*P* < 0.01).

**Fig. 4:**
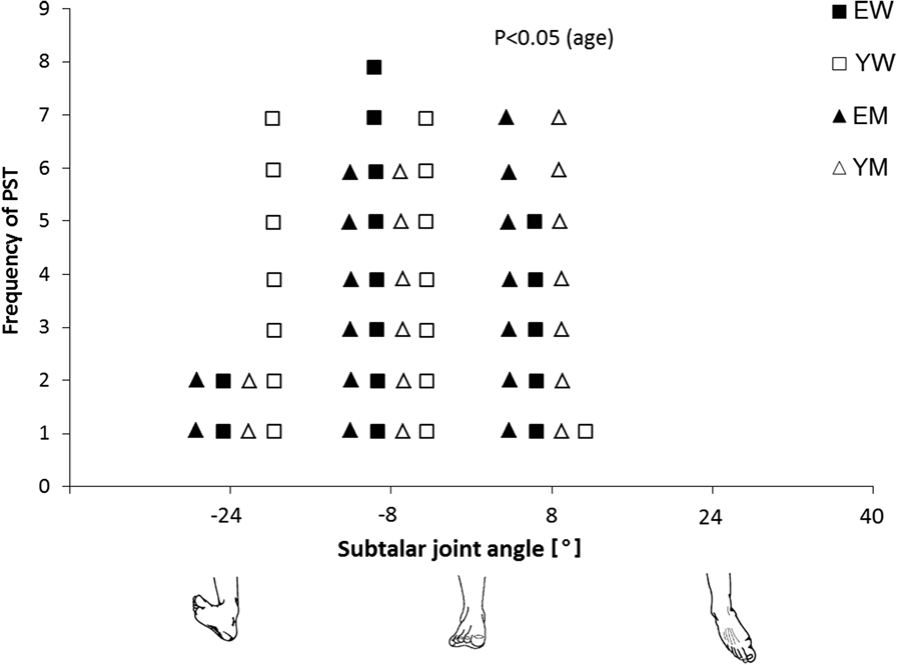
Distribution of the joint angles at which individual subjects attained their greatest peak pronator torque (PPT). EW, elderly women; YW, younger women EM, elderly men; YM, younger men. Chi-square test reveals significant age effect (*P* < 0.05) in angle 8°

In Fig. 4 it is shown how the aforementioned differences are reflected in the chi-square distribution, concerning the subtalar joint angle, at which the peak of the individual pronator strength curve occurs. In younger females, PPT is observed in equal numbers (47 % each) in −24° and −8°. The most frequent PPT angle for elderly females is −8° (53 %), for both younger and elderly males at 8° (47 %). The chi square test reveals a significant difference in frequency of PPT between the sexes at 8°, indicating a shift to the supinated positions for males: only 13 % of the females but 47 % of the males have their PPT at 8°.

**Fig. 5:**
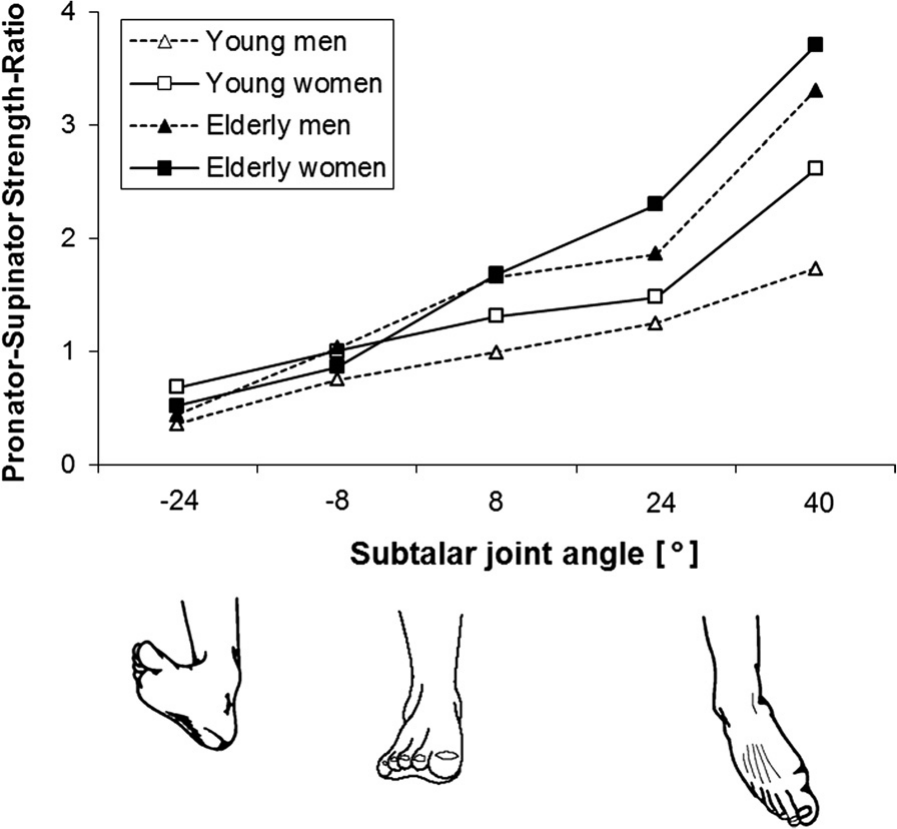
Isometric angle-dependent pronator-to-supinator torque ratio. Mean values are shown without error bars for clarity

The isometric pronator-supinator strength-ratios (PSR, Fig. 5) show an ascending characteristic. Generally, a higher relative supinator strength capacity is found in the pronated positions −24° and −8°, except in elderly males whose PSR is nearly even in −8°. We found higher pronator strength capacity in the supinated positions, except in younger males who show a nearly equalized pronator and supinator capacity in 8°. The ANOVA reveals a significant joint angle x age interaction (*P* < 0.0001; F_(4,56)_ = 5.3; η^2^_p_ = 0.09), indicating a higher angle-specific PSR for the elderly groups. We found significant main effects of joint angle (*P* < 0.0001; F_(4,56)_ = 55.7; η^2^_p_ = 0.5) and age (*P* < 0.01; F_(1,56)_ = 10.1; η^2^_p_ = 0.15) on PSR.

An additional table file shows the descriptive strength data in more detail [see Additional file 1].

## Discussion

The purpose of the present study was to analyse the isometric angle-dependent subtalar pronator and supinator capacity in younger and elderly males and females. The age- and sex-related differences in PST and PPT are in agreement with previous studies whose findings also revealed differences in muscle morphology between males and females [20–23], and between younger and elderly subjects [18, 19].

Consistent with our recent experiment [16], we find descending supinator strength curves from a pronated to supinated position across all investigated groups and inverted U-shaped pronator strength curves in males and elderly females. Significant strength x age interactions can be explained by both differences in muscle activation and muscle-tendon properties. Compared to younger males, elderly males have shown lower muscle volume combined with shorter muscle fibers [30, 31] and reduced pennation angle [32]. These structural differences in muscle architecture are expected to change the angle-torque relationship. In a model, Reeves et al. [30] explained that longer muscle fascicles with a higher number of sarcomeres would cause a greater shortening of each sarcomere for the same whole muscle shortening and, thus, would flatten the angle-torque relationship. Furthermore, tendinous tissue properties have a marked effect on muscle function [32], but the role of these changes in lowered muscle function with aging is not clear as there are inconsistent findings in the area of aging and muscle-tendon complex properties. Aging also reduces agonist activation [33] and increases antagonistic co-activation [34], which both may contribute to age-related differences in the angle-torque relationship.

Significant strength x sex interactions in the strength curves could be caused by sex-differences in the muscle-tendon properties which have been found previously [35]. Data suggest that estrogen may contribute toward a diminished collagen synthesis rate in females, resulting in lower collagen content [36, 37] and reduced ligament stiffness [36]. Significant joint angle x age x sex interactions of pronator strength are related to the strength difference between the younger females and the other three groups in the most pronated position 24°. In this joint angle, young females are able to exert a 41 % higher PPT compared to males (Fig. 2a). Furthermore, our findings reveal a higher pronation ROM and overall subtalar ROM in younger females compared to all other groups which is in agreement with previous research [38, 39]. According to the age x sex interaction in pronation ROM, the large pronator muscle strength of younger females in end-ranged pronation may result from reduced inhibitory tension of the passive joint structures [21–23] which allows them to achieve higher PPTs. One possible explanation for the age x sex interaction in ROM is that estrogen level decreases postmenopausal [36, 37], and given the average age of our older female sample was 66.7 years, this would likely have influenced their results.

An interesting and clinically relevant finding is the lower pronator strength of younger females compared to younger and elderly males in the most supinated position 40°. As pronator strength is crucial to counteract rapid inversion moments, lower pronator strength in this joint angle would be disadvantageous. However, PSR is not different between younger females and younger males. We also found higher relative supinator strength in the supinated angles 8°and 24° for younger males compared to younger females. Both differences in relative subtalar pronator and supinator muscle strength in the supinated positions suggest an angle-dependent lower strength capacity for younger females in relation to their peak torques. Considering the higher incidence rate of lateral ankle sprains in female athletes [40], strengthening the subtalar pronators and supinators in the supinated positions would be beneficial.

To our knowledge, this is the first study documenting the characteristic of the subtalar angle-dependent PSR-curves on the basis of MVIC testing. Previous isokinetic studies analysed the dynamic PSR by dividing the peak pronator torque, which was recorded in a maximum voluntary concentric contraction across the active range of motion, by the peak supinator torque of the subsequent antagonistic contraction [6, 41–46]. By using this procedure, PSR only reflects the ratio of the peak pronator torque to the peak supinator torque neglecting the subtalar joint angle, where the peak torques occur. Consequently, it appears questionable whether this method represents the real PSR. In our opinion, the weakness of this approach could be an explanation for the inconsistent findings of isokinetic assessments of PSR in subjects with chronic ankle instability. In unstable ankles, pronator torque is reported to reach 47 and 84 % of supinator torque during concentric contractions at velocities of 30°/s and 120°/s, respectively [46], while Baumhauer et al. [6] and de Noronha & Borges [44] report on a nearly equalised PSR at 30°/s. In subjects with medial tibial stress syndrome, PSR was found to be about 15 % higher at 30°/s as compared to non-injured controls [45]. In several studies with healthy subjects, pronator torque is reported to reach a value 90 % of supinator torque at 30°/s [6, 42–45]. It was also found that, according to Hill’s force-velocity-relationship, peak pronator and supinator torques will be decreased if testing velocity is increased [6, 43, 46]. Furthermore, this decrease in strength becomes obvious to a lesser extent for supination (−31 %) than for pronation (−44 %) [43]. However, it has not been discussed how this affects PSR. As our findings of isometric strength tests reveal an angle-dependent PSR curve with an increasing shape from the pronated to the supinated joint angles, we recommend isometric measurements in several joint angles to be part of subtalar strength assessment. Isometric subtalar strength testing reflects the real muscular strength capacity when MVICs are performed across the active range of motion [11].

From a clinical point of view, PSR is likely an indicator of muscular imbalance between the medial and lateral stabilisers of the foot. Despite a significant main effect of age and significant age x angle interaction, all groups showed increasing PSR as the foot is moved into greater supination angles. As mentioned, this angle-dependent PSR characteristic, whereby relative pronator strength capacity was higher in end-ranged supination and vice versa, is likely to be advantageous in preventing lateral ankle injuries. However, an appropriate amount of absolute muscle strength is indispensable to counteract external supinator and pronator moments during dynamic movements. Therefore, PSR is a supplementary rather than a single parameter for functional subtalar strength diagnostics.

### Limitations

The likelihood of sustaining an acute ankle injury mainly depends on both foot positioning at ground contact [47, 48] and dynamic joint stabilisation by co-contraction of the surrounding muscles [17]. As dynamic stabilisation is affected by muscle stiffness which is described as the extent to which a muscle resists mechanical stretch [49], one limitation of the present study is that our MVIC testing procedure only reflects the isometric active resulting torque-length-relationship of the pronators and supinators. Further investigations are needed to elucidate the stress–strain characteristics of the passive elements in the subtalar joint movement plane.

A further limitation of the study is that our subtalar testing device has the same axis position for all participants. It has to be mentioned here that variations in the spatial orientation of the subtalar joint axis and other foot axes were found both within the population and in dynamic movements [50].

## Conclusions

When testing the pronator and supinator muscle strength across subtalar range of motion, age- and sex-related differences in subtalar strength profile and range of motion have to be considered as both affect the strength curves and PSR. Younger females were found to have higher pronator strength capacity in the most pronated joint angle, which may bepartly due to their greater subtalar joint range of motion when compared to younger males and elderly subjects. As pronator and supinator muscle strength is important for dynamic joint stabilisation, for both feed-forward control and to counteract inversion and eversion moments, the subtalar strength capacity and the PSR should be assessed isometrically across a wider range of subtalar motion for clinical purposes.

## Additional file

Additional file 1:
**Summary of the strength results of all groups (mean ± SD)**. (PDF 220 kb)

## Abbreviations

MVIC: Maximum voluntary isometric contraction
ROM: Range of subtalar motion
PPT: Peak pronator torque (= maximum resulting voluntary isometric pronator torque)
PST: Peak supinator torque (= maximum resulting voluntary isometric supinator torque)
PSR: Pronator-to-supinator-strength ratio
