# Supplementary material for: Angle-torque relationship of the subtalar pronators and supinators in younger and elderly males and females
Source: J Foot Ankle Res. 2015 Nov 24;8:64. doi: 10.1186/s13047-015-0125-2 (PMC4659193; doi:10.1186/s13047-015-0125-2)
Supplement: Additional file 1: — Summary of the strength results of all groups (mean ± SD). (PDF 220 kb) [file 13047_2015_125_MOESM1_ESM.pdf]

Strength parameter	Subtalar joint angle				
	(-24°)	(-8°)	8°	24°	40°
**Peak supinator torque (Nm/kg)** [A,B,C,D]					
Elderly women	0.35 ± 0.12	0.24 ± 0.10	0.11 ± 0.06	0.06 ± 0.03	0.02 ± 0.01
Younger women	0.60 ± 0.19	0.40 ± 0.12	0.21 ± 0.07	0.11 ± 0.04	0.07 ± 0.07
Elderly men	0.40 ± 0.17	0.30 ± 0.15	0.16 ± 0.07	0.09 ± 0.04	0.04 ± 0.03
Younger men	0.72 ± 0.15	0.50 ± 0.11	0.34 ± 0.08	0.18 ± 0.05	0.10 ± 0.03
**Peak pronator torque (Nm/kg)** [A,B,D]					
Elderly women	0.17 ± 0.12	0.20 ± 0.12	0.17 ± 0.08	0.11 ± 0.05	0.07 ± 0.03
Younger women	0.36 ± 0.13	0.36 ± 0.12	0.26 ± 0.06	0.14 ± 0.03	0.10 ± 0.02
Elderly men	0.17 ± 0.09	0.21 ± 0.09	0.21 ± 0.08	0.13 ± 0.06	0.10 ± 0.03
Younger men	0.25 ± 0.17	0.36 ± 0.08	0.32 ± 0.05	0.22 ± 0.05	0.16 ± 0.04
**Relative supinator strength (%)** [A,C,E]					
Elderly women	100.00 ± 0.00	67.0 ± 16.9	31.90 ± 11.1	16.05 ± 8.6	6.73 ± 4.0
Younger women	100.00 ± 0.00	70.5 ± 23.5	37.58 ± 11.6	18.28 ± 5.6	12.65 ± 12.8
Elderly men	100.00 ± 0.00	70.7 ± 18.9	39.80 ± 12.1	25.53 ± 18.2	12.19 ± 9.6
Younger men	100.00 ± 0.00	69.8 ± 10.1	47.90 ± 8.1	25.92 ± 6.3	14.18 ± 3.9
**Relative pronator strength (%)** [A,C,E,F]					
Elderly women	70.47 ± 22.85	86.4 ± 19.9	76.50 ± 20.1	51.36 ± 20.4	36.09 ± 12.4
Younger women	88.93 ± 16.59	91.9 ± 8.8	67.98 ± 11.4	39.25 ± 13.2	28.13 ± 10.2
Elderly men	71.23 ± 25.71	89.5 ± 13.1	89.69 ± 16.9	59.21 ± 22.1	44.80 ± 15.6
Younger men	60.01 ± 31.28	92.9 ± 7.4	84.36 ± 18.7	59.06 ± 17.1	41.62 ± 10.9
**Pronator-supinator-strength-ratio** [A,B,D]					
Elderly women	0.52 ± 0.44	0.86 ± 0.47	1.68 ± 0.95	2.30 ± 1.32	3.70 ± 1.83
Younger women	0.68 ± 0.37	0.99 ± 0.45	1.31 ± 0.45	1.48 ± 0.45	2.61 ± 2.60
Elderly men	0.44 ± 0.17	1.03 ± 1.00	1.66 ± 1.01	1.85 ± 1.14	3.33 ± 1.96
Younger men	0.36 ± 0.24	0.75 ± 0.19	0.99 ± 0.29	1.25 ± 0.26	1.73 ± 0.68

ANOVA

A Significant main effect: joint angle
B Significant main effect: age
C Significant main effect: sex
D Significant joint angle x age interaction
E Significant joint angle x sex interaction
F Significant joint angle x age x sex interaction
